# Rare Solitary Fibrous Tumor in the Pediatric Neck: A Case Report and Review of the Literature

**DOI:** 10.7759/cureus.1140

**Published:** 2017-04-06

**Authors:** G. Zachary White, Eric L Cox, Erich J Schwartz, Shant A Korkigian

**Affiliations:** 1 Otolaryngology/Facial Plastic Surgery, Beaumont Health- Farmington Hills; 2 Otolaryngology, Michigan State University College of Osteopathic Medicine; 3 Pathology, Beaumont Health

**Keywords:** solitary fibrous tumour, pediatric, pediatric surgery, otolaryngology, pediatric otolaryngology, rare tumor, oncology

## Abstract

Solitary fibrous tumors (SFT) are a rare type of mesenchymal-derived tumor not commonly found in the pediatric population, especially in the head and neck. Tumors of this nature are most commonly seen in the adult population and are identified with unique immunohistochemical markers, specifically signal transducer and activator of transcription 6 (STAT6) and hematopoietic progenitor cell antigen (CD34). Including SFTs in the differential diagnosis while working up a mass can be difficult considering their relatively non-descript appearance on imaging and the low yield immunohistochemical staining that must be ordered to confirm diagnosis. The current literature identifies only a handful of cases of SFTs occurring in the pediatric population, with a majority arising from the pleura. We present the case of a 13-year-old male who underwent radical excision of a left occipital triangle neck mass after radiological and pathological workup failed to conclusively make a diagnosis. Postoperative pathologic analysis revealed it to be an SFT. Due to the exceptionally rare presentation of SFTs in pediatric patients, the aim of this case report is to discuss diagnostic measures, solitary fibrous tumor etiology, as well as a recent risk stratification system used for the evaluation of postoperative disease progression. Our hope is that clinicians will include SFTs in their differential diagnosis when working up a neck mass in the pediatric population.

## Introduction

Solitary fibrous tumors (SFT) are a rare type of mesenchymal-derived tumor with characteristic hemangiopericytoma-like vascular branching patterns that occur throughout the body, including the mediastinum, lung, pleura, liver, kidney, orbit, and meninges [1-3].

These tumors most commonly present in the adult population [3]. Solitary fibrous tumors are exceedingly rare in the pediatric population and create a diagnostic dilemma. Once identified, the treatment of choice for SFTs is radical surgical resection [4]. Given the rarity of SFTs overall, the presenting age of the patient and atypical location of the tumor warrant further discussion in terms of diagnostic measures and case management of pediatric patients presenting with neck masses. The purpose of this paper is to discuss the case of a SFT arising in the left occipital triangle of the neck in a 13-year-old male patient.

## Case presentation

A 13-year-old male was referred to outpatient head and neck surgical oncology for a neck mass presenting in the left occipital triangle. The mass slowly grew over several months and initially presented with some slight irritation during rotation of the neck, in addition to an obvious cosmetic deformity. The patient’s review of symptoms was unremarkable and did not include fever, night sweats, or weight loss. Past medical history and past surgical history were negative. There was no known family history of cancer or lymphoma. Physical exam was otherwise negative. The patient had no accessory nerve or other cranial nerve palsy. The patient had no other neck masses or enlarged lymph nodes present. Laboratory data displayed a normal white blood cell count of 4.6, hemoglobin of 15.2, hematocrit of 46.3, and a platelet count of 272. All electrolyte values were within normal limits.

**Figure 1:**
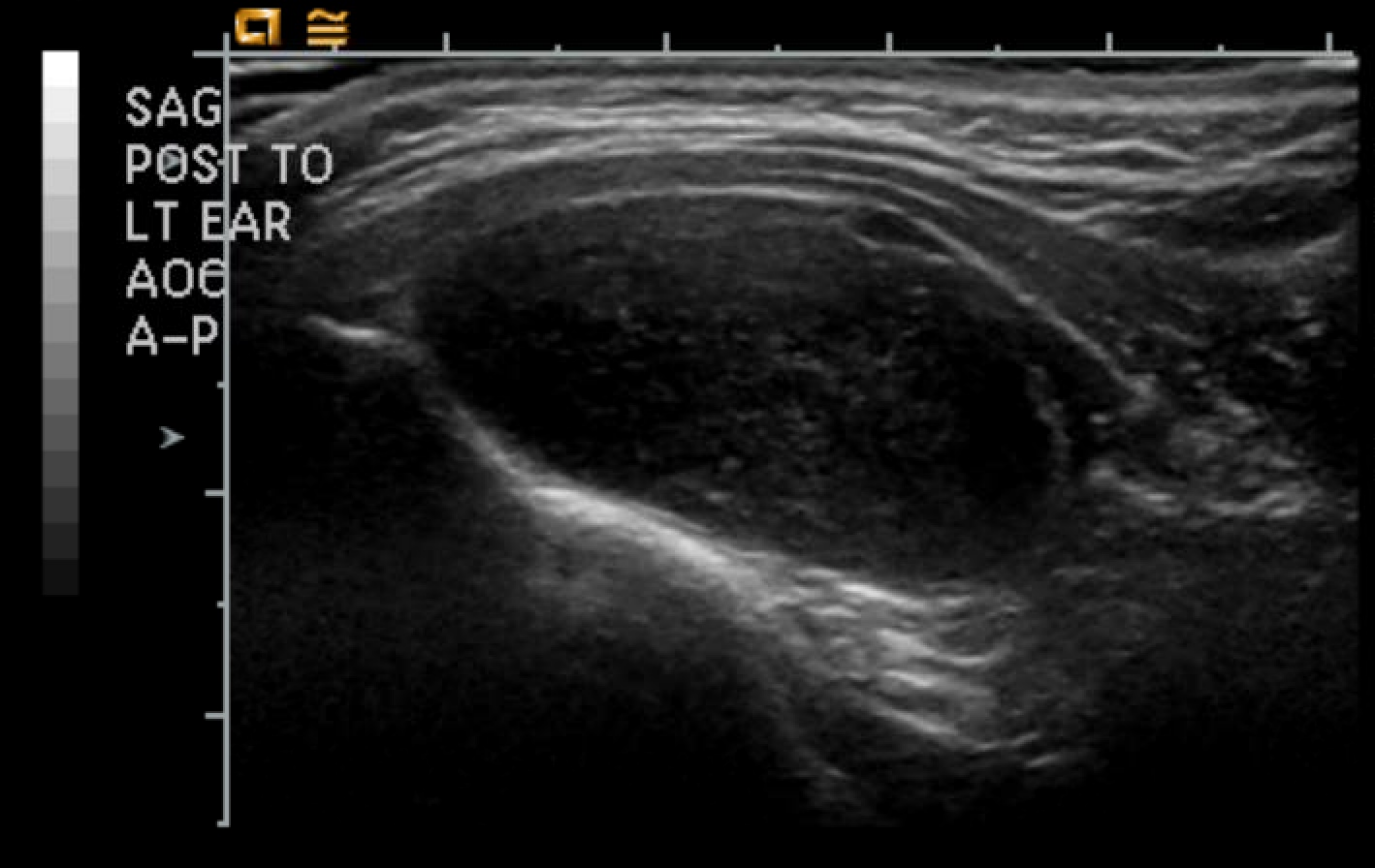
Ultrasound imaging of the solitary fibrous tumor

The differential diagnosis included lipoma, lymphoma, lymphangioma, and malignancy. Ultrasound identified an ovoid, well-circumscribed mass, deep to the muscular layers of the occipital triangle, measuring 4.5 x 3.0 x 1.5 cm in diameter (Figure 1).

No other imaging was performed. A fine-needle aspiration (FNA) yielded inconclusive results, and the decision was made to proceed with surgical removal. Intraoperatively, the mass appeared as a pink-tan to gray lesion that easily broke apart into irregularly shaped fragments, which were homogeneous with focal areas of hemorrhage. The mass, and the associated fragments, were fully removed and the specimen was sent to pathology. The post-surgical pathology, like the previous FNA, initially came back as inconclusive. A sample was then sent to an outside head and neck pathologist for further examination.

**Figure 2:**
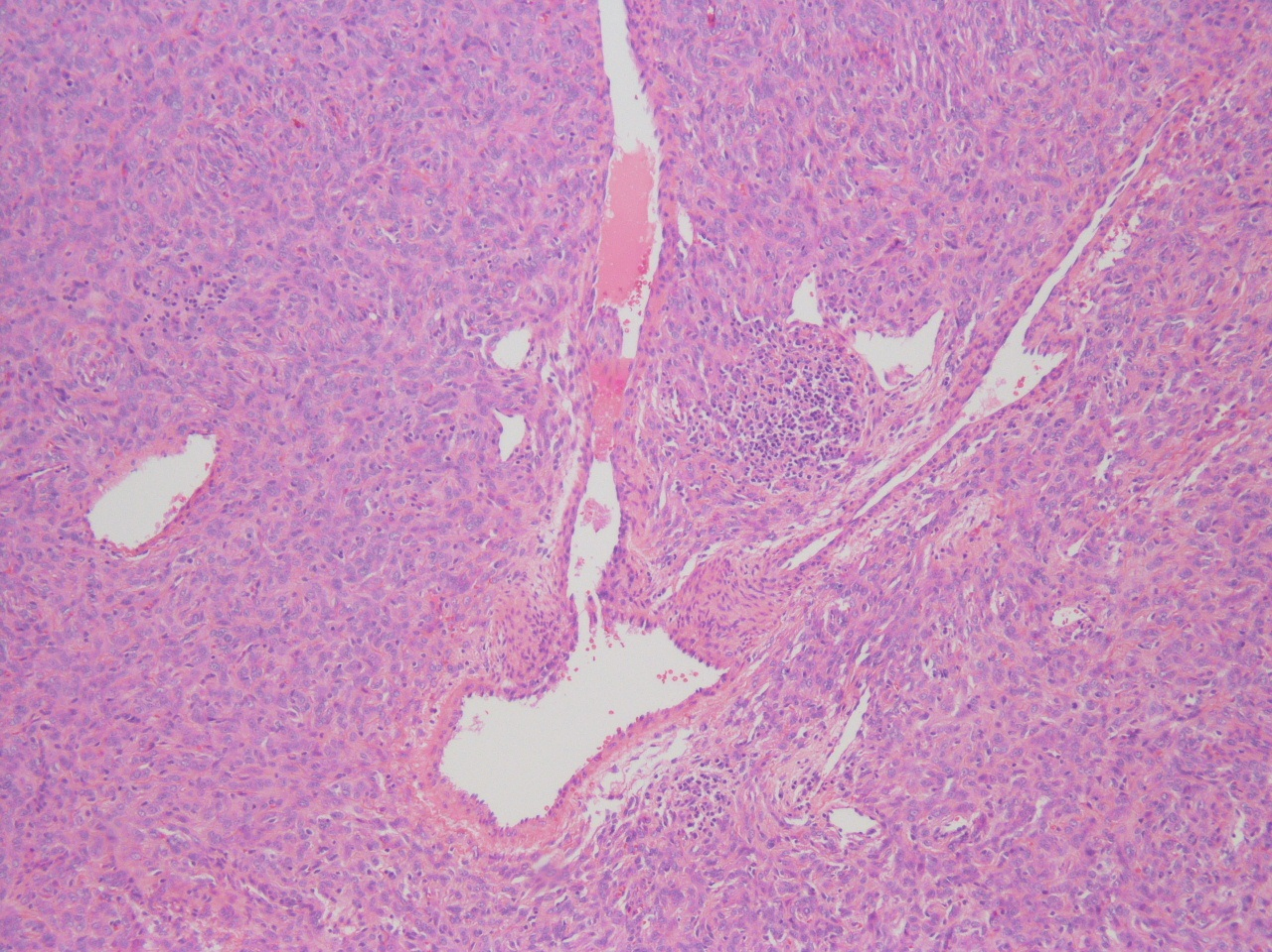
Hematoxylin and eosin (H&E) stain showing staghorn vessels of the solitary fibrous tumor (100x)

**Figure 3:**
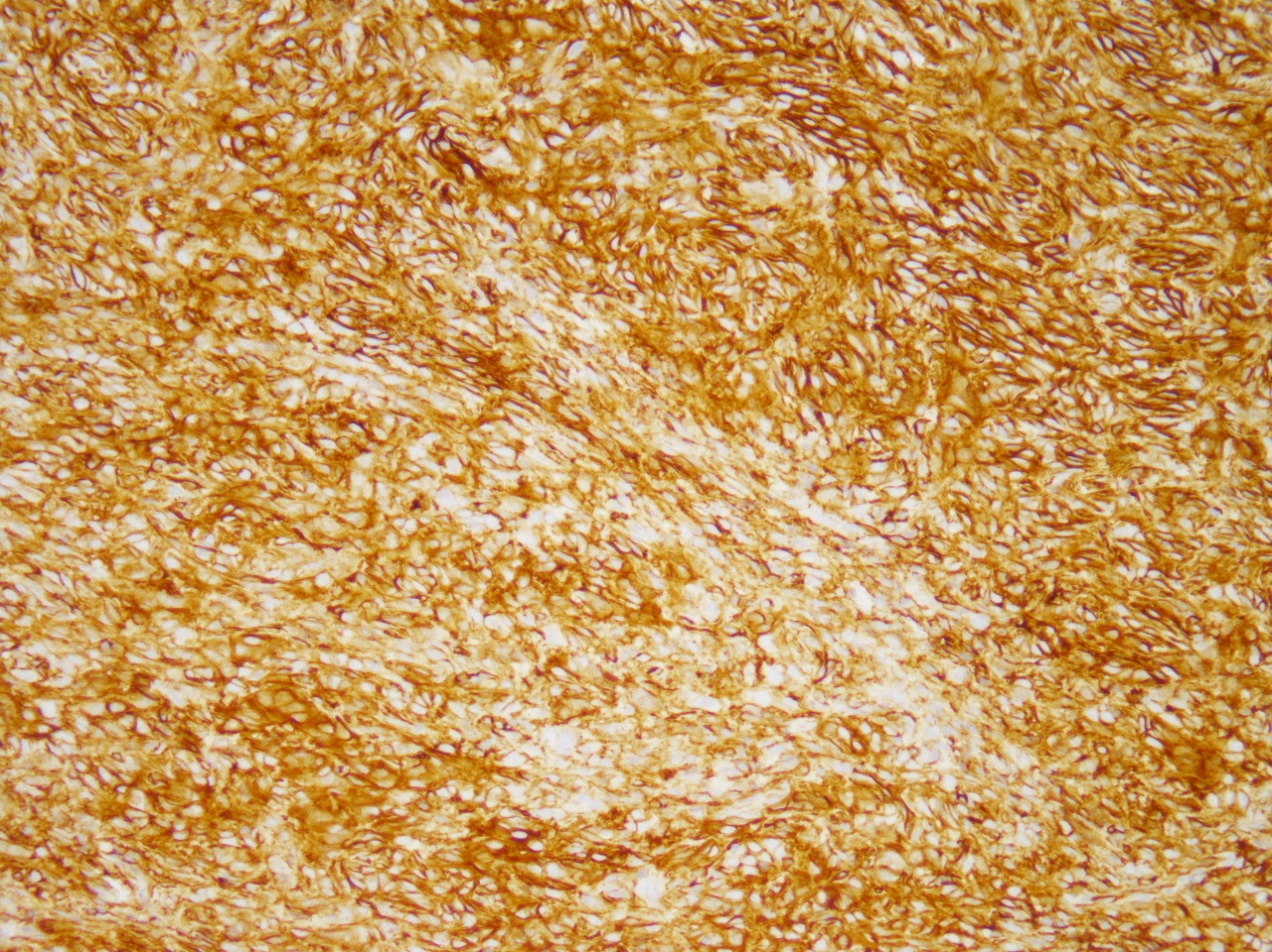
CD34 membranous staining of the solitary fibrous tumor (200x)

**Figure 4:**
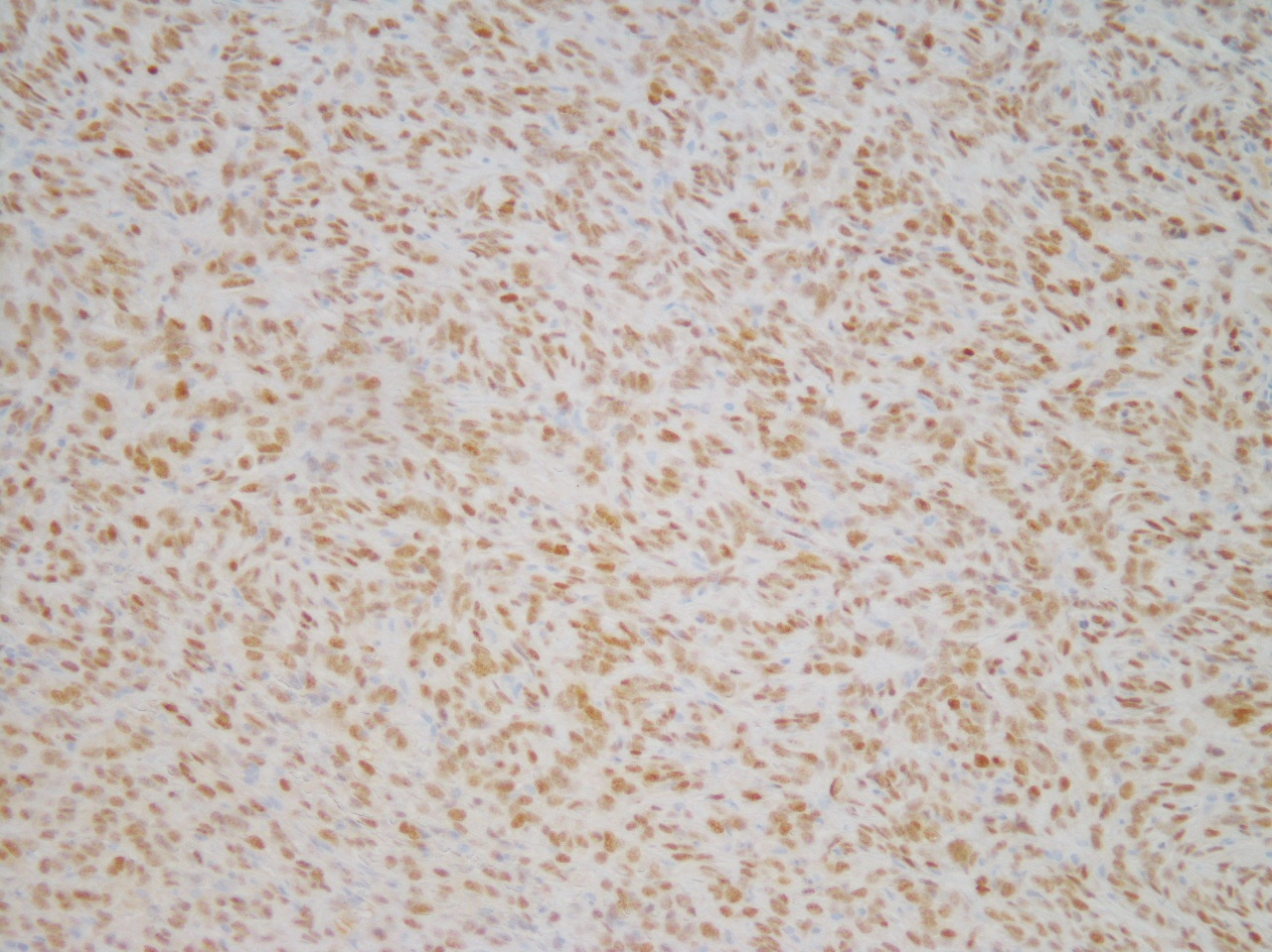
STAT6 nuclear staining of the solitary fibrous tumor (200x) STAT6 - signal transducer and activator of transcription 6

Examination of the soft tissue specimen determined the mass to be consistent with an SFT (Figures 2-4).

Histologic examination showed evidence of cellular spindle cell proliferation with staghorn-type blood vessels. Further immunohistochemical testing indicated that the spindle cells were positive for the cellular markers hematopoietic progenitor cell antigen (CD34) and signal transducer and activator of transcription 6 (STAT6), both consistent with SFTs [2, 5]. The outside pathologist confirmed that the patient was in a low-risk category (0-2 points) for metastasis and recurrence using a recently proposed assessment model for long-term metastasis of solitary fibrous tumors [3]. Despite the low risk for metastasis, there is almost no previous literature detailing with a case of this nature. The decision was made to follow the patient closely with routine examination and radiology as needed. 

## Discussion

Solitary fibrous tumors (SFTs) most often originate in the pleura, with other common sites of occurrence including the extremities, mediastinum, peritoneum, and orbit [4]. The key diagnostic criterion of these tumors is their immunohistochemical reactivity, with CD34 and STAT6 being the most commonly observed cell markers [2,5-6]. A recent study by Doyle, et al. reports that 98% of tumors expressing STAT6 are confirmed to be SFTs [7]. These tumors are rarely malignant, but 10%-15% may metastasize to the lungs, bones, and liver [4].

An English-language literature search provides little information regarding solitary fibrous tumors in pediatric patients. Only two case reports document an SFT arising in the pediatric head or neck: one records an SFT arising in the neck [8] and one case on the scalp [9]. Given the lack of applicable case studies in the extant medical literature, it is difficult to fully assess the risk of malignancy for this patient. Nonetheless, the treatment plan for an SFT in the neck remains the same, as if it presented anywhere else in the body. Surgical resection and subsequent pathological staging, followed by long-term case monitoring, if necessary, is the preferred treatment course [6].

The nature of the patient presentation in this specific case warrants further investigation into diagnostic measures for pediatric patients presenting with neck masses. If the mass is palpable and clearly accessible, then FNA can be used to obtain a preoperative sample. During pathologic evaluation of the suspected SFTs, the most valuable immunohistochemical diagnostic marker has been identified as the presence of CD34 positive spindle cells [4]. Additionally, the cell marker STAT6 has also been shown to be more specific to SFTs, as almost all display the NAB2-STAT6 fusion gene [5]. In conjunction with immunohistochemical evaluation, radiologic studies can also be useful in narrowing down the differential of neck masses. Unfortunately, the index of suspicion for such lesions will inherently be low, given the rarity of these tumors, and therefore, they may be difficult and impractical to identify.

Radiologic findings consistent with SFTs are non-descript, with the mass being described as a well-circumscribed, smooth, and lobulated soft tissue mass on computed tomography (CT) [6]. The tumors tend to enhance homogeneously, but the larger ones may have central tubular or rounded low-attenuation areas, mainly caused by cystic changes or necrosis. A retrospective study did note that the presence of large collateral feeding vessels surrounding the mass as seen on CT might be a distinguishing radiologic feature of SFTs, thus giving credence to the importance of preoperative imaging studies [10]. While not specific, this finding is proposed to help the pathologist narrow the differential diagnosis. While CT scan is not necessarily the first modality for the assessment of a solitary, slow growing, and clinically benign mass in the occipital triangle in the pediatric population, ultrasound gives little insight on the nature of an SFT.

Previous studies have noted that solitary fibrous tumors are typically benign, but there have been reports of recurrence and metastasis, especially with masses exceeding 10 cm in diameter [3, 6]. Previous articles also suggest that surgical excision with complete resection is the most effective initial treatment modality [3]. Stereotactic radiation has also been identified as a beneficial postoperative therapy to effectively control residual tumors [4]. The placement of a radiographic marker at the tumor site intraoperatively could possibly be a measure to ensure that if stereotactic radiation is utilized, the adverse side-effects associated with this treatment could be limited. Thus, radiation treatment could have possibly been explored if the intraoperative sample sent to pathology had not confirmed clean surgical margins.

A risk stratification system has been devised by Demicco, et al., and should be utilized to evaluate for risk of recurrence and metastasis [3]. A score is determined by the patient’s age (< 55, zero points, > 55, one point), tumor size (< 5 cm, zero points; 5-10 cm, one point; 10-15 cm, two points; > 15 cm, three points), and mitotic figures present per 10x high power field (zero figures, zero points; 1-3 figures, one point; > four figures, two points). Through this model, cumulative scores of 0-2 are deemed low risk for metastasis, scores of 3-4 are considered moderate risk for metastasis, and scores of 5-6 place the patient in a high-risk category for metastasis. This risk stratification scale may be used to determine the level of follow-up as well as the treatment modalities for these tumors in adults, but no such literature currently exists in children.

Unfortunately, in our case, even this robust analysis leaves questions to be answered regarding the patient’s metastatic potential, largely in part due to the median age (53-years-old) and range (19 to 83-years-old) of the patients in the study by Demicco, et al. [3]. This being said, while considering the risks of metastasis, however slight, it is wise to continue to observe the patient over the long term. 

The inclusion of SFTs into the diagnostic differential diagnosis for neck masses in the pediatric population may be of greater importance than previously thought. Most present as asymptomatic masses and as such can prove to be a diagnostic challenge [6]. CT and MRI studies have been shown to have some benefit in helping differentiate SFTs from other more malignant neoplasms; they may also be considered in the preoperative management of these cases. The addition of appropriate preoperative imaging studies and conclusive FNA could potentially help identify SFTs quicker. Adding SFTs to the differential diagnosis of lipoma, lymphoma, smooth muscle tumors, nerve sheath tumors, and fibrous histiocytomas allows for a fully comprehensive approach to the management of pediatric neck masses. 

## Conclusions

Solitary fibrous tumors are a rare entity, especially in the pediatric population. Tumors of this nature originate from the pleura and are most commonly present in the adult population. These mesenchymal-derived tumors have characteristic hemangiopericytoma-like vascular branching patterns and stain positive with CD34 and STAT6 immunohistochemical markers. Surgical excision and close monitoring of this tumor in the pediatric population is currently the recommended therapy.
